# Importance of Bladder Radioactivity for Radiation Safety in Nuclear Medicine

**DOI:** 10.4274/Mirt.18480

**Published:** 2013-12-10

**Authors:** Salih Sinan Gültekin, Turan Şahmaran

**Affiliations:** 1 Department of Nuclear Medicine, Dışkapı Yıldırım Beyazıt Training and Research Hospital, Ankara, Turkey

**Keywords:** Radiation protection, Radionuclide imaging, ionizing radiation, radiation effects, radiation monitoring

## Abstract

**Objective:** Most of the radiopharmaceuticals used in nuclear medicine are excreted via the urinary system. This study evaluated the importance of a reduction in bladder radioactivity for radiation safety.

**Methods:** The study group of 135 patients underwent several organ scintigraphies [40/135; thyroid scintigraphy (TS), 30/135; whole body bone scintigraphy (WBS), 35/135; myocardial perfusion scintigraphy (MPS) and 30/135; renal scintigraphy (RS)] by a technologist within 1 month. In full and empty conditions, static bladder images and external dose rate measurements at 0.25, 0.50, 1, 1.5 and 2 m distances were obtained and decline ratios were calculated from these two data sets.

**Results:** External radiation dose rates were highest in patients undergoing MPS. External dose rates at 0.25 m distance for TS, TKS, MPS and BS were measured to be 56, 106, 191 and 72 μSv h-1 for full bladder and 29, 55, 103 and 37 μSv h-1 for empty bladder, respectively. For TS, WBS, MPS and RS, respectively, average decline ratios were calculated to be 52%, 55%, 53% and 54% in the scintigraphic assessment and 49%, 51%, 49%, 50% and 50% in the assessment with Geiger counter.

**Conclusion:** Decline in bladder radioactivity is important in terms of radiation safety. Patients should be encouraged for micturition after each scintigraphic test. Spending time together with radioactive patients at distances less than 1 m should be kept to a minimum where possible.

**Conflict of interest:**None declared.

## INTRODUCTION

Nuclear medicine staff and others who have close contact with radioactive patients are exposed to external radiation at various degrees. Although numerous radiopharmaceuticals in nuclear medicine have been used for diagnostic imaging studies, 99m-technetium (99mTc)-labeled agents are the most commonly used in routine nuclear medicine. In order to obtain target organ specific imaging, radiopharmaceuticals are generated by the combination of a radionuclide with a bioactive agent such as methylene diphosphonate (MDP), methoxy isobutyl isonitrile (MIBI), or diethylenetriamine pentaacetic acid (DTPA). The urinary system is the main excretion route for most of the 99mTc-labeled radiopharmaceuticals used in nuclear medicine (1,2).

Radiation dose or dose rates per application and in total to staff within a time period such as daily, monthly or annually can be determined by two different methods (3,4,5,6,7,8). At first, it is calculated by measurements obtained at a fixed distance from the patient and some authors also take into consideration the time spent with the patient at a constant distance to calculate the amount of expected radiation exposure to technicians (4,5). In the other method the calculation is based on the direct reading of data obtained from the electronic pocket dosimeter of radiation workers (6,7,8).

In this study, conducted in a center performing routine nuclear medicine tests, an evaluation was made on the potential contribution of the decrease in bladder radioactivity after voiding to radiation safety.

## MATERIALS AND METHODS

The study group consisted of 135 patients (75 males, 60 females, age range: 21-85 years, mean age: 51±14 years). Scintigraphic studies were performed on all 135 patients within a 1-month period by the same technologist. This study was approved by the Local Ethics Committee of Dışkapı Yıldırım Beyazıt Training and Research Hospital and informed consent was obtained from the patients.

Thyroid scintigraphy (TS) in 40/135 patients, whole-body bone scintigraphy (WBS) in 30/135 patients, myocardial perfusion scintigraphy (MPS) in 35/135 patients and renal scintigraphy (RS) in 30/135 patients were carried out using a large field of view dual-head gamma camera (E-cam, Siemens, USA). For TS, a neck image was obtained at the anterior, right and left oblique positions at the 15th minute following intravenous (i.v) injection of 185 MBq (5 mCi) 99mTc-pertechnetate. Then, an anterior static image of the bladder region was taken before and after voiding. For WBS, anterior and posterior whole-body images were taken 3 hours after i.v injection of a 925 MBq (25 mCi) dose of 99mTc-MDP. In addition, an anterior static image of the bladder was obtained from each patient immediately after micturition. For the MPS, rest and stress scans were performed with one-day protocol. After i.v injection of 99mTc-MIBI with a 296 MBq (8 mCi) dose for the rest study and a 888 MBq (24 mCi) dose for the stress study, images were taken at 60 minutes and 30 minutes respectively after the injection. Following routine imaging, anterior static images of the bladder before and after voiding were taken. For RS, a 370 MBq (10 mCi) dose of 99mTc-DTPA was injected intravenously under the camera and dynamic imaging was performed for 40 minutes; at the 20th minute 1 mg/kg furosemide was administered intravenously. At the end of the routine imaging, anterior static images of the bladder were obtained for the full and empty bladder. On the anterior static images, a standard rectangular region of interest (ROI) of 2x2 cm was drawn on the bladder region for before (ROIb) and after (ROIa) voiding.

External radiation dose rate (EDR), simultaneous with the scintigraphic imaging, was measured at 0.25, 0.5, 1, 1.5 and 2 m distances from the skin at the level of the bladder before (EDRb) and after (EDRa) voiding using a Geiger counter (Ludlum 14C model, Pancake GM external detector, Ludlum Ins., USA). Calibration of this device was done in the Turkish Atomic Energy Agency, Nuclear Research and Training Center, Second Grade Standard Dosimetry Laboratory.

Decline ratio in radioactivity for scintigraphic and Geiger studies was calculated for all patients. This ratio shows the % difference between the measurements obtained with the full and empty bladder. The calculations were made using following formulas: [100 - ROIa / ROIb x 100] for scintigraphic measurements and [100 – EDRa / EDRb x 100] for Geiger measurements. 

## RESULTS

**Figure 1:**
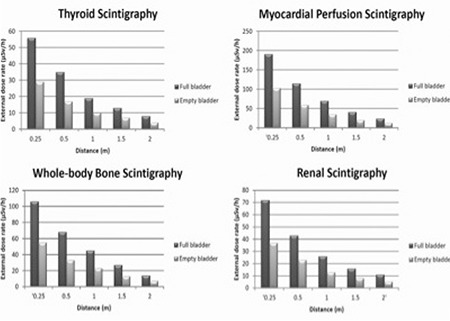
Comparison of average radiation dose rate measurements obtainedfor full and empty bladder status at 0.25, 0.50, 1, 1.5 and 2 m frompatients undergoing thyroid scintigraphy, whole-body bone scintigraphy,myocardial perfusion scintigraphy and renal scintigraphy

Figure 1 presents the mean values of the EDR measurements obtained at 0.25, 0.5, 1, 1.5 and 2 m distances in patients with a full and empty bladder for various scintigraphic examinations, including TS, WBS, MPS and RS.EDR values in each scintigraphic test tend to decrease in direct proportion with the increasing distance and bladder emptying. EDRs were the highest at 0.25 m distance in patients with a full bladder undergoing MPS (191 µSv h-1) compared to the others (106 µSv h-1 for WBS, 72 µSv h-1 for RS and 56 µSv h-1 for TS). EDR values were significantly higher at distances under 1 m when compared with the distances greater than 1 m. For example, for WBS in patients with a full bladder, EDR values were measured as 106 µSv h-1 at 0.25 m and 68 µSv h-1 at 0.5 m while the values were 27 µSv h-1 at 1.5 m and 14 µSv h-1 at 2 m.

**Figure 2:**
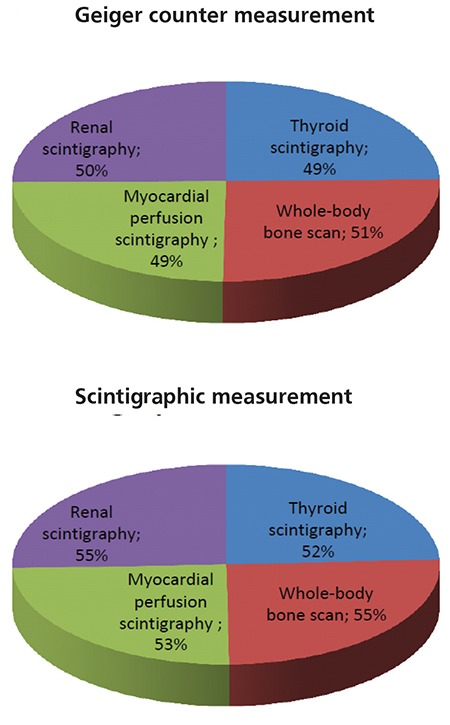
The percentage distribution of the total average decline ratiosin radiactivity according to the type of scintigraphic examinations for themeasurements obtained from Geiger counter and scintigraphic method

Table 1 shows the average decline ratios in radioactivity between full and empty bladder for 0.25, 0.5, 1, 1.5 and 2 m distances. Average decline ratios range between 46% and 59% without any obvious effect of distance and scintigraphy type. In addition, Figure 2 demonstrates the total average decline ratios according to the scintigraphic tests for Geiger counter measurements and scintigraphic measurements. Although total decline ratios for the values obtained with the scintigraphic method are slightly higher than the values from the Geiger counter, the results obtained by both methods still support each other. As a result, these findings have shown that a reduction of about 50% in external radiation exposure via disposal of bladder radiation could be achieved.

## DISCUSSION

Some authors have stated that external radiation exposure from radioactive patients is a matter of priority for radiation safety of nuclear medicine staff and the public (7,9). Technologists are frequently exposed to external radiation while performing procedures such as preparing and administering radiopharmaceuticals, positioning the patients under the gamma camera, data acquisition, removing the patients from the bed and accompanying the radioactive patient (3). The received external radiation dose can be reduced by various measures (3,7,10). Several authors have studied external radiation doses and protection methods (3,4,5,6,7,8,10,11).

Bayram et al. (3) found that 99mTc-MIBI MPS gave higher doses to technologists when compared with other scintigraphies using ^99m^Tc-labeled agents. Chiesa et al. (4) reported that the measured dose range for most typical scintigraphic tests was within 0.2-0.4 µSv except for two; equilibrium angiocardioscintigraphy (1.0+/-0.5 µSv) and 99mTc-MIBI single-photon emission computed tomography (1.7+/-1.0 µSv). Clarke et al. (6) reported that an average radiation dose experienced by a technologist was 1.5 μSv per scintigraphic study for routine procedures other than cardiac studies, and was a total of 5.5 μSv (rest;1.0 μSv, exercise;2.5 μSv and pharmacological; 2.0 μSv) for cardiac procedures with ^99m^Tc-MIBI or tetrofosmine. Culver et al. (10) calculated the average monthly dose to nuclear medicine technologists by whole-body film badge readings and they found that it was significantly higher for ^99m^Tc-MIBI than 201Tl (450 µSv vs. 100 µSv, p < 0.001). In addition, 360 µSv was reported for noninvasive cardiology staff. The findings of the current study concur that MPS using 99mTc-MIBI causes higher EDR than other traditional scintigraphic studies with 99mTc-labeled agents. Greaves and Tindale (11) measured the maximum dose rate at a distance of 0.1 m for MPS using 99mTc-MIBI. This value was found to be 391.74 µSv h-1 for the one-day protocol and 121.84 µSv h-1 for the two-day protocol. In the current study, in the full bladder state, a dose rate of 498 µSv h-1 was measured at 0.25 m for 99mTc-MIBI study in the one-day protocol. Gomes-Palacios et al. (5) stated that the maximum dose from MPS with a double injection of 99mTc-MIBI was 499 µSv and there was no probability of staff receiving a higher dose than the permissible limits. Lundberg et al. (8) found that doses to the technologist were 5.4 µSv/d and 1.4 mSv/y for scanning and these levels were much lower than the regulatory limits. These findings were consistent with the findings of this study.

Smart et al. (7) found that the usage of a 0.5 mm lead apron for gated stress MPS with ^99m^Tc-MIBI resulted in a significant decrease (0.58±0.56 µSv vs. 1.10±0.45 µSv, 47%) in radiation dose exposure for the technologist. In a study by Bayram et al. (3) the ranges of deep-dose equivalent for technologists from routine scintigraphic examinations (TS, WBS, MPS and RS) were 0.13±0.05 to 0.43±0.17 μSv with a lead shield and 0.21±0.07 to 1.01±0.46 μSv without a lead shield, with a decrease in the range of 38% - 57%. In this study, it was demonstrated that a significant decline in radiation exposure, equivalent to the use of a protective lead apron, could be obtained by effective micturition of radioactive patients. This decline was determined to be in a range between 52% and 55% according to the scintigraphic measurements and between 49% and %51 according to the Geiger counter measurements. We found that median values of the radiation dose rates measured at various distances were the values measured at 1 m distance, and the mean values were between the values measured at 0.5 and 1 m distances. To reduce the radiation exposure of radiation workers at distances less than 1 m, we suggest that staff should carry out their work related to radioactive patients carefully and as quickly as possible while remaining within professional limits.

## CONCLUSION

Reduction in bladder radioactivity after voiding is a practice that provides a significant decrease in radiation exposure for the staff and any others in the vicinity of the patient. Patients should be encouraged to go to the toilet before and after scintigraphic tests to achieve a higher level of radiation safety. Spending time together with radioactive patients at distances less than 1 m should be kept to a minimum whenever possible.

## Figures and Tables

**Table 1:**
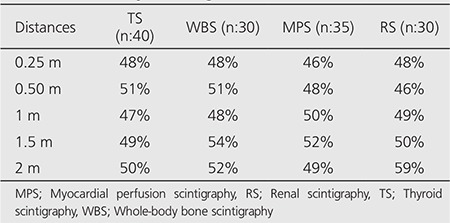
The average decline ratios in bladder radioactivitycalculated from the radiation dose rates measuredin full and empty bladder at 0.25, 0.5, 1, 1.5 and2 m distances by the Geiger counter
